# On the Reproducibility of Label-Free Quantitative Cross-Linking/Mass Spectrometry

**DOI:** 10.1007/s13361-017-1837-2

**Published:** 2017-12-18

**Authors:** Fränze Müller, Lutz Fischer, Zhuo Angel Chen, Tania Auchynnikava, Juri Rappsilber

**Affiliations:** 1 Chair of Bioanalytics, Institute of Biotechnology, Technische Universität Berlin, 13355 Berlin, Germany; 2 Wellcome Trust Centre for Cell Biology, School of Biological Sciences, University of Edinburgh, Edinburgh, Scotland EH9 3BF UK

**Keywords:** Quantitation, Cross-linking, Human serum albumin, Label-free, Mass spectrometry, Reproducibility

## Abstract

Quantitative cross-linking/mass spectrometry (QCLMS) is an emerging approach to study conformational changes of proteins and multi-subunit complexes. Distinguishing protein conformations requires reproducibly identifying and quantifying cross-linked peptides. Here we analyzed the variation between multiple cross-linking reactions using bis[sulfosuccinimidyl] suberate (BS^3^)-cross-linked human serum albumin (HSA) and evaluated how reproducible cross-linked peptides can be identified and quantified by LC-MS analysis. To make QCLMS accessible to a broader research community, we developed a workflow that integrates the established software tools MaxQuant for spectra preprocessing, Xi for cross-linked peptide identification, and finally Skyline for quantification (MS1 filtering). Out of the 221 unique residue pairs identified in our sample, 124 were subsequently quantified across 10 analyses with coefficient of variation (CV) values of 14% (injection replica) and 32% (reaction replica). Thus our results demonstrate that the reproducibility of QCLMS is in line with the reproducibility of general quantitative proteomics and we establish a robust workflow for MS1-based quantitation of cross-linked peptides.

**Graphical Abstract:**
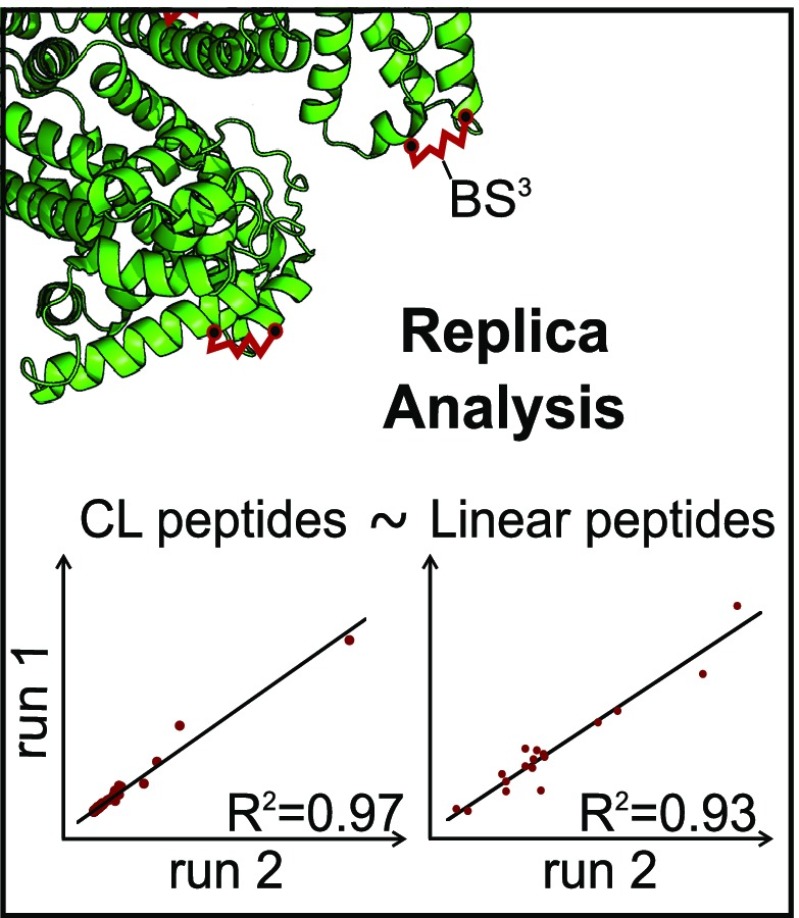
ᅟ

ᅟ

## Introduction

Cross-linking/mass spectrometry (CLMS) has become a powerful tool aiding the structural analysis of proteins and their complexes [1–5] since its onset almost two decades ago [6, 7]. Reaction with a cross-linker converts 3D proximity of amino acid residues into covalent bonds. The bridgeable distance between residues depends on the cross-linker used. Bis[sulfosuccinimidyl] suberate (BS^3^), one of the most commonly used reagents, links residues up to 25–30 Å apart (Cα-Cα distance) [1]. Following proteolytic digestion of the proteins, cross-linked peptides are identified using liquid chromatography-mass spectrometry (LC-MS) and database search.

Previous studies have used CLMS to investigate the structures of single proteins [8], multi-protein complexes [9], and protein–protein interaction networks [10, 11]. The proteins in these studies are often undergoing dynamic conformational changes, which are difficult to determine and visualize by knowing only the sites of cross-linking. For this, understanding the dynamics through relative abundances of certain cross-linked residue pairs is required by adding quantitation to CLMS pipelines. In mass spectrometry-based proteomics there are two broad quantitative strategies, label-free and labeled approaches, both of which are suitable for CLMS. A previous study by Huang 2006 [12] using an ^18^O labeling-based QCLMS approach had several drawbacks that prevented widespread use of this approach, including incomplete labeling and inadequate software for data analysis. Fischer et al. 2013 [13] overcame these hurdles by using an isotope-labeled cross-linker and developing the software tool XiQ, which combined the accuracy of manual peak validation with the convenience of automated quantitation. Since then, several software packages became available to analyze QCLMS data [14, 15]. Although isotope labeling-based QCLMS has been used successfully in several studies [14, 16–19], it suffers from the usual limitations that often come with the experimental design of labeling approaches: cost of isotope-labeled reagents (which can be expensive), complex sample preparation, and reduced data coverage [20, 21]. In contrast, label-free quantitation can avoid these pitfalls and there are no limits to the numbers of samples that can be compared. Advantages of label-free quantitation were presented recently with an MS2-based QCLMS workflow using Skyline [22]. A general caveat of label-free approaches is that samples are processed separately, which can result in technical biases during sample preparation [21]. As the sample preparation procedure of cross-linking is more elaborate than in normal proteomics, one might expect a larger variance.

Here we investigate the reproducibility of label-free QCLMS. We determined the variation introduced during sample preparation and contrast this with the variation between multiple injections during LC-MS acquisition. As a model system, we cross-linked human serum albumin (HSA) using bis[sulfosuccinimidyl] suberate (BS^3^) and we adapted Skyline into a workflow for semi-automated label-free QCLMS.

## Methods

### Reagents

HSA was purchased from Sigma Aldrich (St. Louis, MO, USA). The cross-linker BS^3^ was purchased from Thermo Scientific Pierce (Rockford, IL, USA).

### Cross-Linking Reaction

Ten cross-linking reactions were performed in parallel as follows: purified human serum albumin (40 μg; 2 μg/μL) in cross-linking buffer (20 mM HEPES-KOH, pH 7.5, 20 mM NaCl, 5 mM MgCl_2_,) was mixed with BS^3^ (160 μg, 30 μg/μL in cross-linking buffer) and cross-linking buffer (14.6 μL), to a total reaction volume of 40 μL (1 μg/μL protein concentration) with a protein to cross-linker mass ratio of 1:4. After 1.5 h incubation on ice, the reaction was stopped using 5 μL saturated ammonium bicarbonate (~2.5 M) for 30 min at room temperature. Forty μg of cross-linked HSA from each reaction were subjected to SDS-PAGE and protein bands were visualized using Coomassie staining. Cross-linked HSA monomer bands were excised for digestion.

### Sample Preparation for Mass Spectrometric Analysis

**Figure 1:**
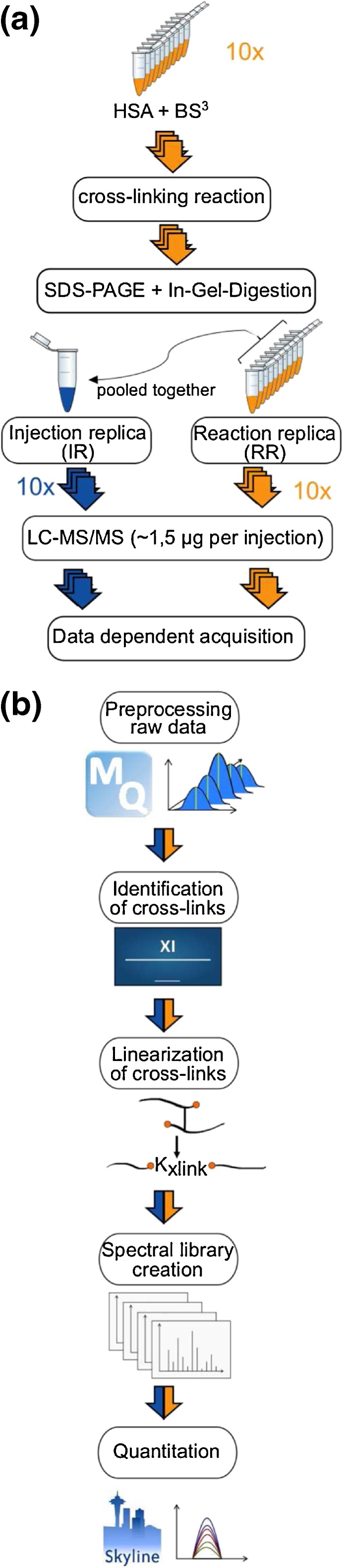
Label-free cross-linking quantification workflow. (**a**) Workflow for sample preparation: orange represents reaction replica and blue injection replica. (**b**) Workflow for cross-link identification and quantification using MaxQuant for peak picking, Xi for cross-link identification and Skyline for quantification

Each sample-containing gel band was digested separately [23]. Proteins were reduced with 10 mM dithiothreitol, subsequently alkylated with 55 mM iodoacetamide, and then digested using trypsin (300 ng/μL). After digestion, peptides were extracted using 80% v/v acetonitrile (ACN) in 0.1% v/v trifluoroacetic acid (TFA). Tryptic peptides were desalted using C_18_-Stage Tips [24] and eluted with 80% v/v ACN, 0.1% v/v TFA prior to mass spectrometric analysis. Peptides were concentrated in a Vacufuge Concentrator (Eppendorf, Germany) and resuspended in 2% v/v ACN, 0.1% v/v formic acid (FA) to a final protein concentration of 0.75 μg/μL; 4/5 (nominally 32 μg) of each reaction sample was pooled as injection replica. The remaining 1/5 (nominally 8 μg) of each reaction sample was used for reaction replica experiment. Nominally for each mass spectrometric acquisition, 1.5 μg peptides were injected (Figure 1a).

### LC-Mass Spectrometric Analysis

LC-MS/MS analysis was performed using Orbitrap Fusion Lumos (Thermo Fisher Scientific, CA, USA) with a “high/high” acquisition strategy. The peptide separation was carried out on an EASY-Spray column (50 cm × 75 μm i.d., PepMap C_18_, 2 μm particles, 100 Å pore size, Thermo Fisher Scientific, Germany). Mobile phase A consisted of water and 0.1% v/v FA and mobile phase B consisted of 80% v/v ACN and 0.1% v/v FA. Peptides were loaded onto the column with 2% buffer B at 0.3 μL/min flow rate and eluted at 0.25 μL/min flow rate with following gradient: 150 min linear increase from 2% to 40% mobile phase B followed by 11 min increase from 40% to 95% mobile phase B. Eluted peptides were sprayed directly into the mass spectrometer and analyzed using a data-dependent acquisition (DDA) mode. In each 3 s acquisition cycle, precursor ions were detected in the Orbitrap with resolution 120,000 and *m/z* range 400–1600. Ions with charge states from 3+ to 7+ were selected for fragmentation. The selection priority was set to first lowest charge and then highest intensity. Selected ions were isolated in the quadrupole with a window size of *m/z* 2. The isolated ions were fragmented by high energy collision dissociation (HCD) and analyzed with resolution 30.000 in Orbitrap. Dynamic exclusion was enabled with the exclusion duration set to 60 s and exclusion mass tolerance was set to 10 ppm.

### Identification of Cross-Linked Peptides

The raw mass spectrometric data files were processed into peak lists using MaxQuant [25] (v. 1.5.0.0). “FTMS top peaks per 100 Da” was set to 20, “FTMS de-isotoping” box was unticked, and all other parameters were set to default (Figure 1b). The subsequent database search was conducted using Xi [26] against the sequence of HSA (UniProt ID: P02768) with the reversed HSA sequence as decoy. The following search parameters were used: MS accuracy: 6 ppm, MS/MS accuracy: 20 ppm, enzyme: trypsin, missed cleavages: 4, cross-linker: BS^3^, fixed modification: carbamidomethylation on cysteine, variable modification: oxidation of methionine and modification by BS^3^ with the second NHS ester hydrolyzed or aminated. The BS^3^ reaction specificity was assumed to be at lysine, serine, threonine, tyrosine, and the N-termini of proteins. The data have been deposited to the ProteomeXchange [27] Consortium via the PRIDE [28] partner repository with the data set identifier PXD007250 For all identified cross-links that were auto-validated by Xi Software, the Cɑ-Cɑ distance between cross-linked residue pairs was measured in the crystal structure of HSA (PDB: 1AO6 chain A). Residue pairs with distance ≥ 30 Å and cross-links matched to decoys were excluded from subsequent quantitation using Skyline.

### Creation of Spectral Library for Autovalidated Cross-Links and Quantitation Using Skyline

**Figure 2:**
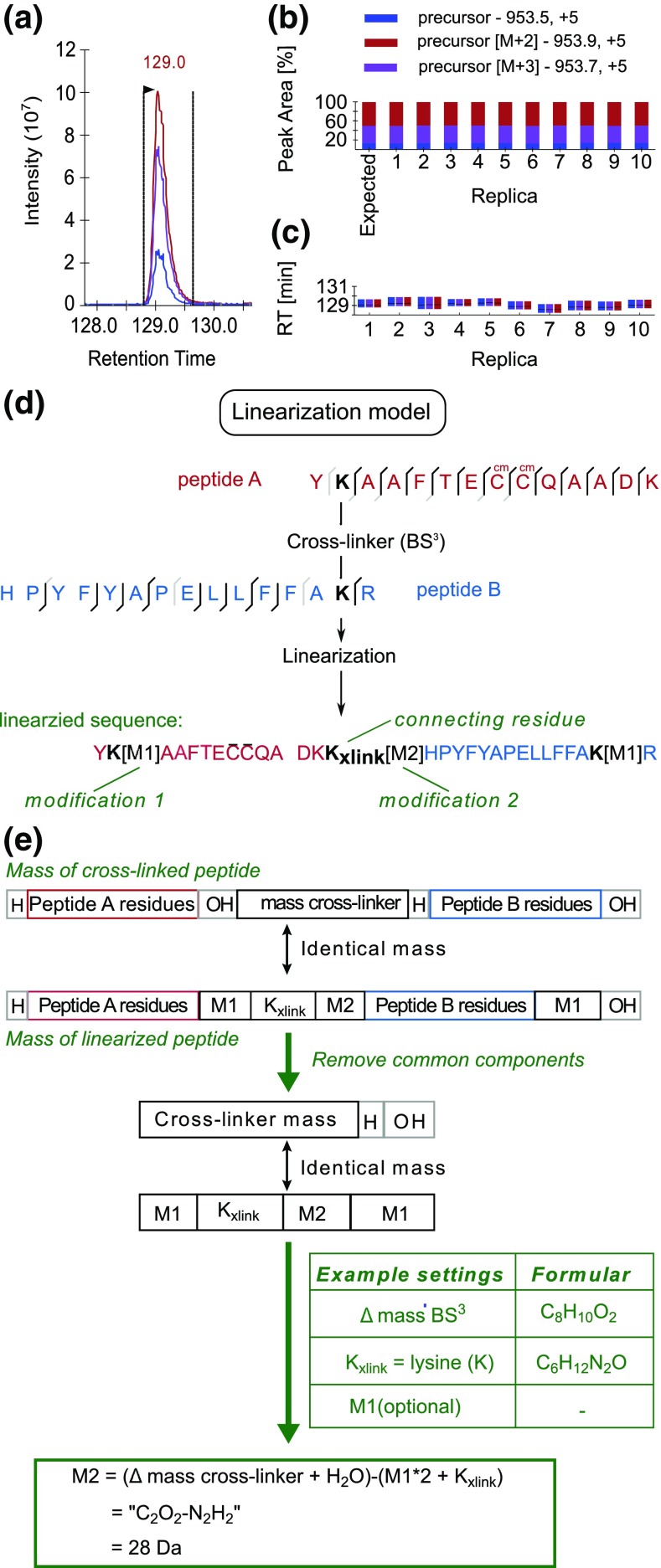
Cross-linked peptides in Skyline. (**a**) Chromatogram view of a linearized cross-linked peptide, showing the MS1 extracted ion chromatogram for the precursor isotope ions M (blue), M+1 (purple), M+2 (red). (**b**) Peak areas, after integration and normalization, of each replica (1–10) with summed up isotope peaks. (**c**) Retention time [min] comparison between all 10 replicas for the presented peptide with apex of the peak (black middle line). (**d**) An example showing the scheme of converting the sequence of a BS^3^ cross-linked peptide into a linear form in format of Skyline input. (**e**) The scheme of mass calculation for linearization of cross-linked pep ide sequences

Quantitation was performed on MS1 level using Skyline (ver. 3.5) [29]. The identification information of cross-linked peptides was introduced as an .ssl file following the standard format for custom libraries in Skyline [30]. The .ssl file is constructed using an in-house script [31] based on the list of peptide spectrum matches (PSM) of identified cross-links. In the .ssl file, an entry is generated for each cross-linking feature. A cross-linking feature is defined as a unique PSM for a cross-linked peptide with differences in charge state, linkage sites, or modification. Since Skyline does not natively support cross-linking data, the sequences of cross-linked peptides were converted into their linear forms, based on the principle described in Chen et al. 2016 and Maiolica et al. 2007 [19, 23] (Figure 2d and e). Skyline uses the .ssl file and the assigned mzML files (created from raw files using MSconvert [32]) to create a spectral library by BiblioSpec. Peptide settings were as follows: enzyme: trypsin KR/P, max missed cleavages: 9, minimal length of peptide: 6, maximal length: 60, modifications: carbamidomethylation on cysteine, oxidation on methionine, cross-linker (lysine + 27.983 Da), BS^3^-OH (156.078 Da), BS^3^-NH2 (155.094 Da) and BS^3^-loop (138.068 Da). Transition settings were set to: precursor charges: 3–7; ion type: p (precursor); mass range: *m/z* 400–1600; tolerance: *m/z* 0.055; isotope peaks included: count 3; mass analyzer: orbitrap; resolution: 120,000 at *m/z* 200. For the remaining settings the defaults were used. MS1 filtering was done as described in the Skyline Tutorial (ver.2.5 [33]). Skyline uses the spectral library to detect so-called transitions of identified precursors. The transitions of a single precursor consist of the intensity measurements across multiple MS1 spectra of selected isotopic peaks of the precursor. For each precursor the peak areas of transitions are integrated and interpreted as quantification signal. After automated peak picking and retention time alignment of Skyline, a manual correction of wrong peak boundaries was performed. Data from Skyline was exported into a .csv file for further processing. Concerning peak areas, Skyline is able to calculate a coefficient of variation (CV), which was used to compare the reproducibility of quantification within experiments. CVs represent the mean variation of peak areas within 10 replicas to determine the variation introduced either by mass spectrometry or by conducting experiments in parallel. The CV was compared for 10 injection replicas and 10 reaction replicas separately. For each of them, the CV value of a cross-linked feature was calculated by Skyline. Furthermore, the CV value for a cross-linked residue pair was calculated as the median of CVs of all cross-linked features that are corresponding to this residue pair.

## Results and Discussion

### Data Quality

To assess the reproducibility of quantitative CLMS in a label-free experiment, we measured cross-linked HSA, a well-studied model protein for CLMS [13], to monitor reproducibility of 10 cross-linking reactions and 10 LC-MS injections of the same sample. HSA was cross-linked in solution using BS^3^ and digested in gel using trypsin. Unfractionated peptides were analyzed by LC-MS using a “high-high” (Orbitrap MS1 and MS2) acquisition strategy and data-dependent acquisition (DDA).

**Figure 3:**
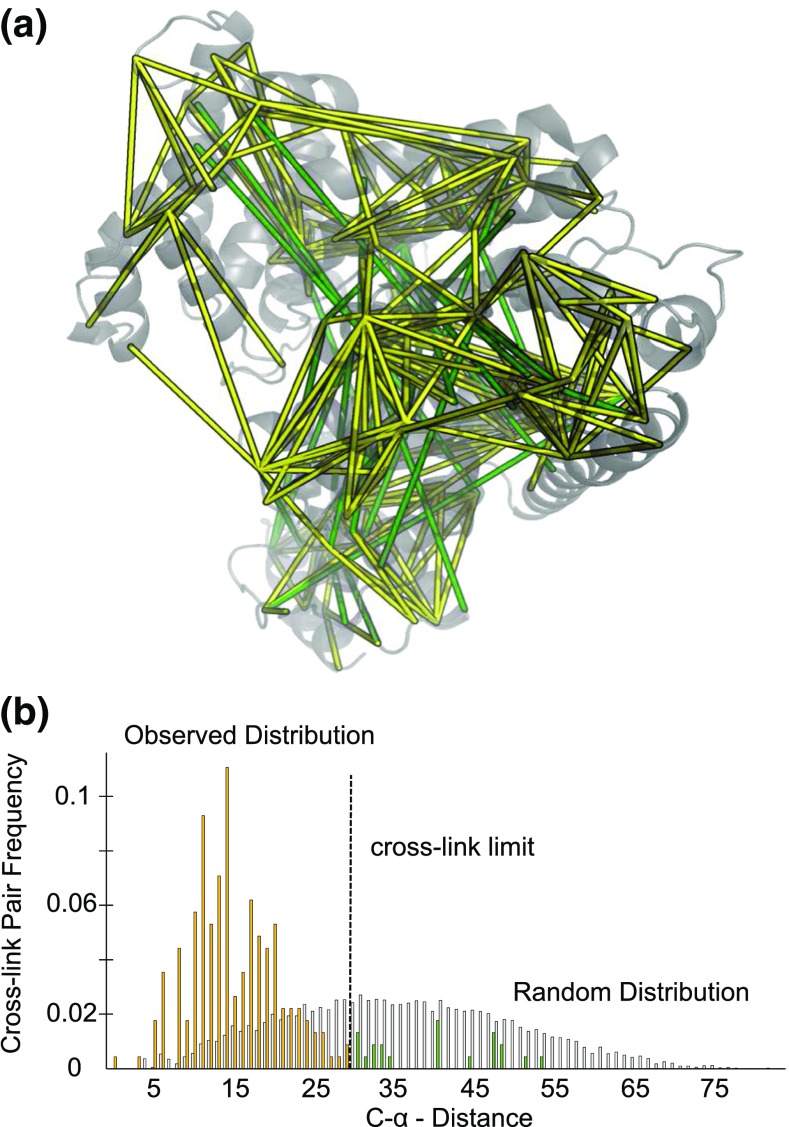
Data quality of identified unique residue pairs using autovalidation in Xi (n = 242, incl. five decoys, i.e., <2% FDR). (**a**) Crystal structure of HSA (PDB:1AO6, chain A) containing identified cross-links (yellow: cross-links within the cross-link limit of BS^3^ n = 221, green: long distance cross-links (≥ 30 Å) n = 21, incl. five decoys). (**b**) Cα distance distribution of observed links (yellow, green) and a random distance distribution (grey)

A cross-linked peptide was considered identified if it passed the auto-validation implemented in Xi without any further manual validation. Combining all injection and reaction experiments resulted in 242 identified unique cross-linked residue pairs (Figure 3a). Compared with the crystal structure, 21 out of these 242 cross-link distances were over the theoretical 30 Å limit of BS^3^ [13] and removed from further analysis to increase the confidence in our data (Figure 3b). Thus, our final set of cross-links included 221 unique cross-linked residue pairs of HSA. This compares favorably with previous studies cross-linking HSA with the same chemistry, which found 43 [13] and 101 [34] intra-protein links with an FDR of 5%.

### Identification of Cross-Linked Peptides by Xi

**Figure 4:**
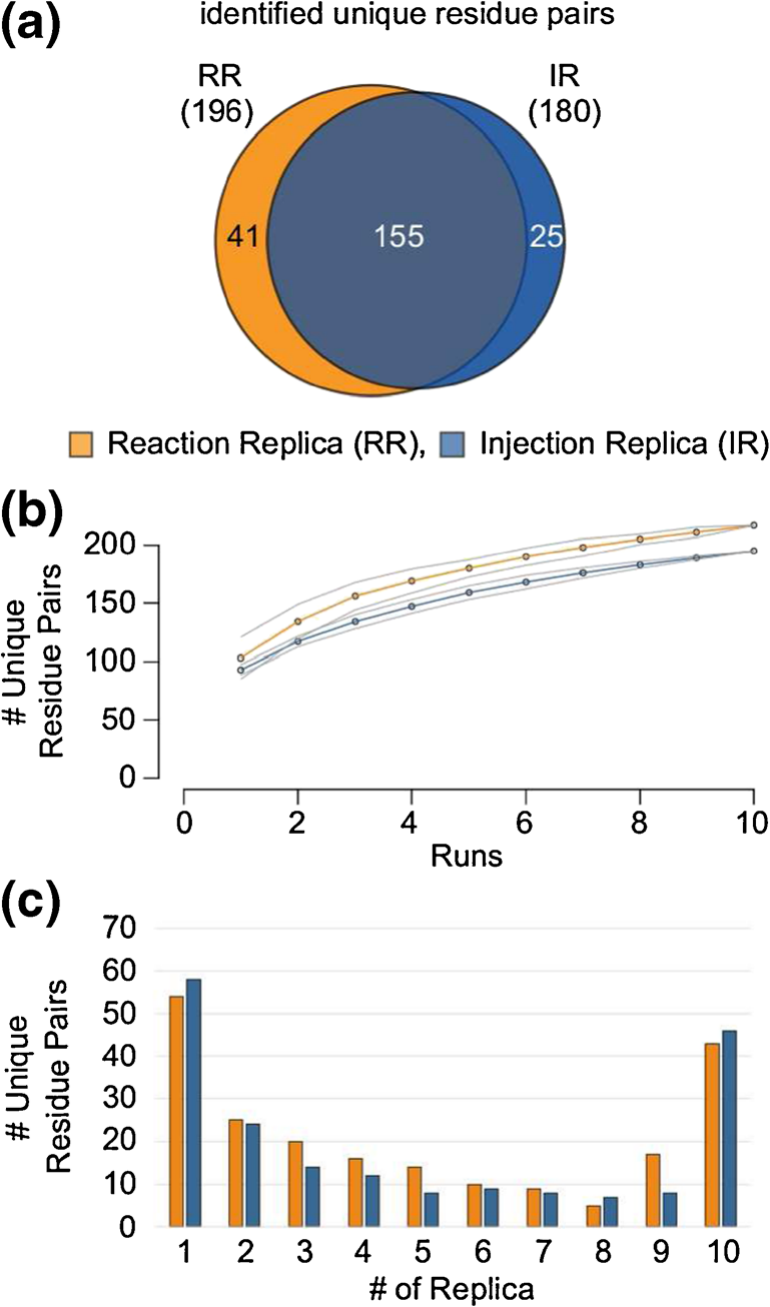
Reproducibility of identification of cross-links in reaction and injection replica. (**a**) Venn-diagram showing overlap in identified residue pairs from reaction (orange) and injection replica (blue). (**b**) The number of identified unique residue pairs are plotted against the number of LC-MS runs, showing the saturation on number of identified residue pairs with increasing number of runs (orange: reaction replica, blue: injection replica, grey: standard deviation). (**c**) Number of unique residue pairs against number of replicas, showing in how many replicas a given unique residue pair was observed (orange: reaction replica, blue: injection replica)

The 10 reaction replicas (RR) yielded in total 196 unique residue pairs whereas the injection replica (IR) yielded 180 unique residue pairs, with 155 (RR: 80%, IR: 86%) common to both datasets (Figure 4a). On average, triplicate analyses yielded an additional 51% cross-linked residue pairs for RR and 47% for IR compared with a single run. This broadly matches observations in recent studies of linear peptides [35–37]. The additional gain of identified residue pairs drops with increasing number of replicas. Adding three reaction replicas to the initial three replicas resulted in a further gain of 22% (IR: 25%), and three additional replicas to the initial six added 11% (IR: 13%) (Figure 4b). Fifty percent of the total number of identifications from 10 replicas could be achieved with two replicas. Triplicates return 2/3 of the total for 10 replicas (RR: 72%, IR: 69%) and might constitute a good compromise between coverage and measurement time. In agreement with this, few links were identified in all 10 replicas (RR: 43 links, 20%; IR: 45 links, 23%) and a sizable fraction of the total links was seen only once (RR: 53, 25%; IR: 58, 30%) (Figure 4c). These results are in accordance with random sampling in DDA experiments [37]. Note that the number of quantified residue pairs is larger, attributable to match between runs.

### Label-Free Quantification of Cross-Linked Peptides by Skyline

For label-free quantification, we used Skyline [29]. In short, we prepared a spectral library comprising all 196 identified residue pairs (1064 spectra) from the reaction replica experiment and all 180 identified residue pairs (885 spectra) from the injection replica experiment. For each experiment, every identified cross-linked peptide can in principle be quantified across 10 replicas even if it was not identified in all of them by DDA.

Prior to quantitation in Skyline, we created a Skyline input file (.ssl file) for each experiment using an in-house script. The .ssl file contains the following information for each identified cross-linked peptides: the assigned mzML file, the scan number, charge state, sequence (including modifications), score type, and score. In this file, the sequence of each cross-linked peptide has been converted into a linear representation with an additional modified lysine residue connecting two linked peptides A and B (K_xlink_, Figure 2d) [19], giving rise to an identical mass to the original cross-linked form (Figure 2e). Skyline used the .ssl file and the assigned mzML files to create a spectral library using BiblioSpec. Peptide and transition settings had to be defined to explore the library and import peptides that matched the filter settings or the library into the quantitation worksheet.

**Figure 5:**
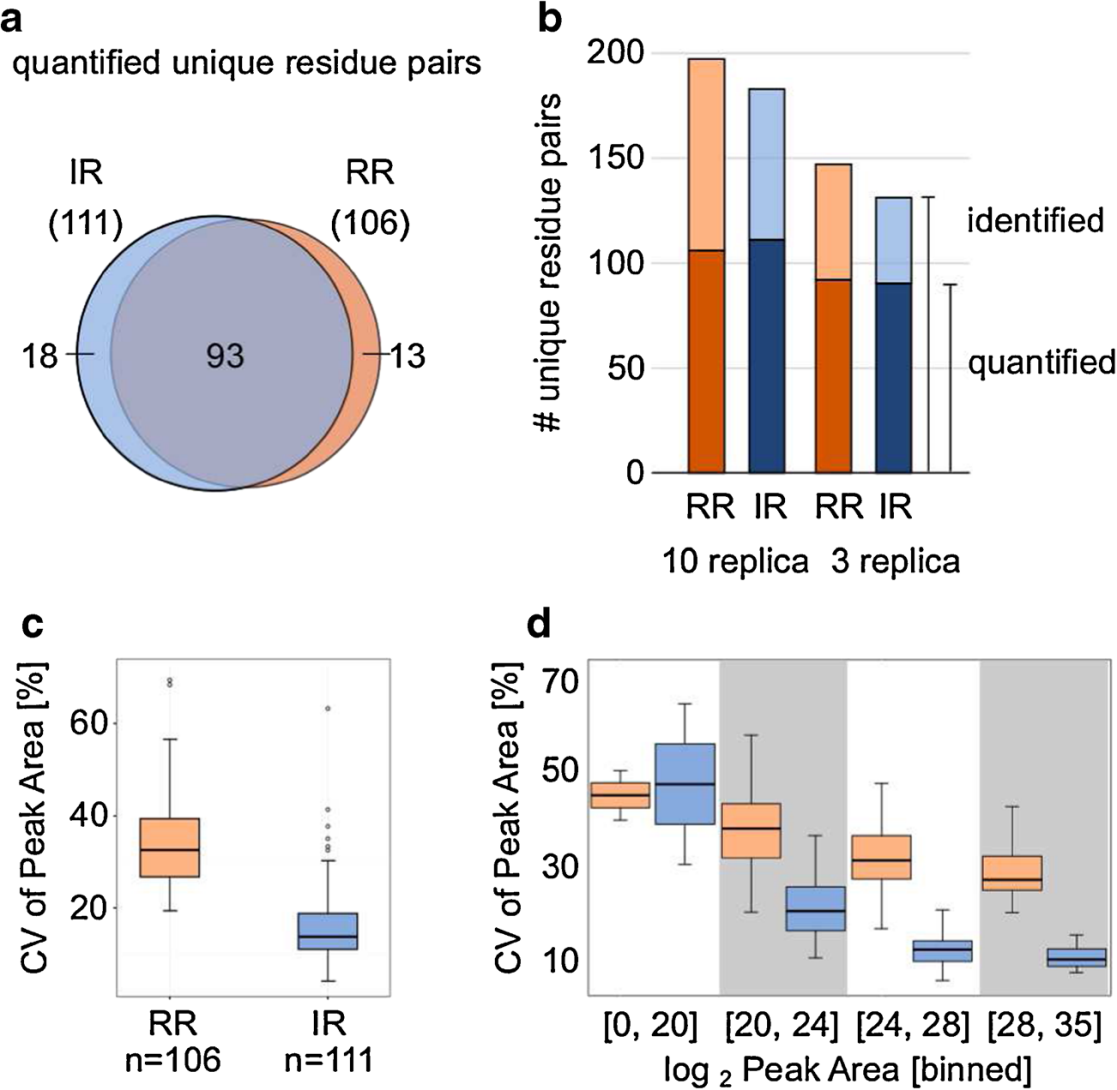
Reproducibility of residue pair quantitation. (**a**) Venn diagram showing number of quantified residue pairs from reaction replica (orange) and injection replica (blue). (**b**) Number of identified (light color) and quantified (dark color) residue pairs (orange: reaction replica, blue: injection replica). (**c**) Coefficient of variation (CV) from median peak areas in % for each experiment after quantification, showing the reproducibility of label-free quantification using cross-linked peptides. (**d**) CV from binned log_2_ peak areas in %, showing anticorrelation between residue pair peak areas and CV values. Reaction replica in orange and injection replica in blue

To increase the confidence of our quantitation results, we excluded peptide pairs from our dataset that were observed with alternative residue pairs if these were not fully separated in the LC dimension (IR: 18, RR: 10 residue pairs). To simplify the evaluation task, we included only cross-linked residue pairs that were quantified across all 10 replicas. This resulted in 106 and 111 quantified unique residue pairs for the reaction and injection experiment, respectively. Most cross-links that were seen and quantified in one set of replica were also seen by the other (93 residue pairs), suggesting that these links are the most abundant (Figure 5a). Many proteomic studies are designed starting with three reaction replica. Here, using three reaction replicas instead of 10 replicas increased the ratio of quantified to identified cross-links from 106 out of 196 (54%) to 92 out of 146 (63%) (Figure 5b). Note that decreasing the number of replicas reduces the number of identified residue pairs from 196 to 146. This is comparable to other studies dealing with quantitative cross-linking [14].

To assess the reproducibility of peak area after quantitation on unique residue pairs, we calculated the coefficient of variation (CV) of a residue pair as the median CV values of all corresponding cross-linked peptide features. The CV values of quantified features are calculated in Skyline, representing the mean variation between peak areas of all replicas. The higher the value the more variation exists between the peak areas over all replicas. As expected, injection replica resulted in higher reproducibility (CV 14%) than reaction replica (CV 32%) (Figure 5c). Simply injecting 10 times from the same tube carries higher reproducibility than starting 10 cross-link reactions in parallel. There is no general consensus on what CV constitutes a good basis for quantitative statements. However, the results fit into variations observed in other studies [35, 37–43]. Perrin et al. 2013 [41] assessed quantitative label-free approaches of linear peptides using cerebrospinal fluid in terms of injection reproducibility and inter-individual variation. Most of the quantified proteins showed a very low coefficient of variation (<5%) for injection replica, which is remarkably low, and a much higher variance across samples from different individuals (48%). Our lower injection reproducibility might be explained by having many modified cross-linked peptides (methionine oxidation and alternative cross-link products) and early eluting peptides, all of these being a source of technical variability. Kramer et al. 2015 [42] reported an injection variance of 10% and an inter-assay variability of 16% using label-free quantification of proteins and data-independent acquisition (DIA). Lai et al. 2015 [43] suggested to use a CV of 30% as threshold for injection replica to get reproducible quantifications using label-free approach and data-dependent acquisition (DDA) strategy.

Finally we investigated reproducibility (CV) in relation to median peak area of residue pairs (Figure 5d). Quantitation reproducibility is linked inversely with peak intensity, as one would expect. Reaction replicas show less reproducibility than injection replicas, but the intensity dependence of reproducibility remains present. Lowering abundance increases variation and reduces reproducibility of quantitation. One should therefore inject as much material as feasible. In summary, the reproducibility of quantitative CLMS and studies with linear peptides are very comparable.

## Conclusion

In this study, we demonstrate that cross-linked residue pairs are identified with reproducibility and saturation characteristics that resembles random sampling in standard shotgun proteomics [37]. Additional injections improve the number of identifications but also increase variability between runs caused by random sampling. Hence, a reliable quantitation procedure when seeking quantitative information is needed. We described a quantitative cross-linking workflow based on DDA and label-free quantitation in Skyline. This allows leveraging of information from multiple injections due to matching features and identifications between runs. We observe that label-free quantitation in cross-linking is in line with the reproducibility of studies using linear peptides. Quantitative cross-linking has already proven its potential for structural and mechanistic studies of proteins and reliable label-free quantitation of cross-linked residue pairs now offers a set of new avenues and experimental designs.
